# Intimate partner violence during pregnancy and risks of low birth weight and preterm birth in hospitals of Tigray, Northern Ethiopia

**DOI:** 10.1038/s41598-024-51569-8

**Published:** 2024-01-16

**Authors:** Kahsay Zenebe Gebreslasie, Solomon Weldemariam, Gelawdyos Gebre, Dawit Zenebe, Mihreteab Mehari, Aklil Birhane, Rebecca Susan Dewey

**Affiliations:** 1 Department of Midwifery, College of Health Science, Mekelle University, Mekele, Ethiopia; 2 Department of Epidemiology, Institute of Public Health, Mekelle University, Mekele, Ethiopia; 3 Department of Nursing, College of Health Science, Mekelle University, Mekele, Ethiopia; 4 Sir Peter Mansfield Imaging Centre, University of Nottingham, Nottingham, UK

**Keywords:** Risk factors, Signs and symptoms

## Abstract

Intimate partner Violence (IPV) can affect any woman, irrespective of their economic status, religion, or culture. This is a human-rights issue and due to its prevalence and adverse effects on pregnancy and birth, it must be given greater attention. Further, there is a lack of data in the Tigray region about adverse birth outcomes due to intimate partner violence during pregnancy. The aim of this study was to assess intimate partner violence during pregnancy and its association with low birth weight and preterm birth in Tigray region. Across-sectional study design was used. 647 women were involved in the study. Simple random sampling techniques were employed to select health facilities and systematic sampling was used to select study participants. Data were entered using Epi info version 3.5.1 and was analyzed using SPSSversion 20. Logistic regression analysis was conducted to assess the association between exposure to intimate partner violence during pregnancy and preterm birth and low birth weight while adjusting for possible confounders. The prevalence of intimate partner violence during pregnancy was 7.3% and the prevalence of low birth weight and preterm birth were 18.5% and 10.8% respectively. There was a statistically significant association between exposure to intimate partner violence during pregnancy and low birth weight. After adjustment for socioeconomic status, women’s habits and obstetric factors, the pregnant women who were exposed to intimate partner violence during pregnancy were two times more likely to have a child with a low birth weight (2.39 (95% CI: 1.26–4.55)). The prevalence of intimate partner violence during pregnancy, low birth weight, and preterm birth in this study was high. Women who experienced intimate partner violence during pregnancy had an increased risk of low birth weight. These findings justify a call to the federal minster of health to take measures aimed at avoiding intimate partner violence during pregnancy to reduce adverse birth outcomes.

## Introduction

Intimate partner Violence (IPV) can occur in all groups of women irrespective of economic status, religion, or culture^1^. Currently it is a human rights issue, and due to its prevalence and adverse effects on birth outcomes, attention has been given to this topic worldwide^2^. Globally, 35% of women have experienced physical and or sexual intimate partner violence or non-partner sexual violence^3^. In Ethiopia, women experiencing IPV ranged from 23% experiencing physical violence to 10% experiencing sexual violence, with 4% of pregnant women facing physical violence^4^. Clinical studies from Africa have also reported that 3% of women experienced sexual violence and 49% experienced psychological violence during pregnancy^2^.

Women who were physically or sexually violated by their partner have a greater than 16% risk of giving birth to a low birth weight baby and have twice risk of abortion and depression, compared to their counterparts^3^. Low birth weight and preterm birth are the main predictors of neonatal mortality and morbidity. Globally, 15–20% of babies are born at a low birth weight, which represents 20 million birth per year^5^. An analysis across thirty studies from Ethiopia indicated that 17.3% of babies are delivered with low birth weight^6^. As consequences of low birth weight, newborn exposed to mental retardation, hypothermia, physical problem and hypoglycemia^6^.

More than 10% of babies are born preterm worldwide^7^. More than one million children die each year secondary to preterm birth^7^ and it is the main cause of mortality in the under-fives^8^ and the burden is more found in less developed countries and also affected both the life of mother and children^9^. Similarly in Ethiopia, from the total number of babies delivered, 10% of them are premature^9^.

Studies have shown that experiencing IPV during pregnancy increases the risk of low birth weight^10–12^. Similarly, some studies have indicated that IPV during pregnancy has a significant association with preterm birth^11,12^, while other studies have shown no such association^13^. There is lack of data on describing the rates of IPV during pregnancy in Ethiopia as a whole, and no studies have been reported in the Tigray region. Moreover, the association between IPV and the rates of low birth weight and preterm birth has not been investigated in the study area, and this study aims to fill this gap. The findings from this study may also be used as baseline for further study in the area.

Since violence is a problem globally, and low birth weight and preterm birth are the leading causes of neonatal mortality and morbidity, this study aimed to address the research gap by characterizing the rate of IPV during pregnancy and quantifying its association with low birth weight and preterm birth.

## Methods

### Study setting and design

The Tigray region is located 783 km from Addis Ababa, capital city of Ethiopia. According to the 2007 census, the population was estimated to be 4,316,988. Women of childbearing age (15–49) comprised 251,650 of this population. According to the 2015 Tigray regional health bureau annual report**,** the area hosts a total of one specialized hospital, 15 general hospitals, 22 primary hospitals, 204 health centers, 712 health posts, and three private hospitals. There are 51 special doctors, 87 general practitioners, 3092 nurses, and 792 midwives in the region. This study utilized an institution-based cross-sectional design. The study was conducted from February-June 2018.

### Inclusion and exclusion criteria

Women who gave birth in selected hospitals of the Tigray region and who were present during the data collection period were included in the study. Babies with visible congenital anomalies and unknown gestational age were excluded from the study.

### Study participants

Participants were postpartum women that gave birth in Tigray region healthcare institutions, together with their newborn babies. The sample size was calculated based on a previous study. A study from Ethiopia reported the rate of intimate partner violence during pregnancy to be 25.8%^14^. Based on this figure, together with a 95% confidence interval, a 5% margin of error, a design effect of 2, and an expected non-response rate of 10%, the required sample size was calculated to be 648. Within the Tigray area, there are 41 hospitals that provide a delivery service, comprising 1 specialized hospital, 15 general hospitals, 22 primary hospitals, and 3 private hospitals. Healthcare facilities were stratified into private and public hospitals, and 1 private hospital and 8 public hospitals were selected using a simple random sampling technique. Participants from each selected healthcare facility were selected using systematic sampling, whereby every third postpartum woman would be included in the sample until the required sample size has been reached. If the selected woman was not eligible to be a participant, the next woman would be considered. The required number of facilities and rate of recruitment was estimated by considering the average numbers of clients expected to deliver daily during the data collection period, i.e., the previous daily client flow of each unit was obtained by referring to the client registration book/record for a month prior to data collection. It was estimated that three months' recruitment at the average client load as measured proceeding the data collection period would be sufficient, and this was used to allocate the sample size proportionally to each of the selected healthcare facilities.

### Data collection tool

A structured questionnaire was prepared in English by reviewing the published literature. The English questionnaire was translated to Tigrigna (local language) by a language expert and back translated to English to maintain internal consistency. Data collection was conducted through interviewer-administered techniques. To ensure the validity of the questionnaire expert review was made.

Outcomes of interest for this analysis pertained directly to neonatal outcomes, which were obtained by a chart review within 48 h of delivery. Birth weight (g) and gestational age (weeks) were taken directly from the chart.

### Operational definition

Low birth weight was assigned if the neonate weighed < 2500 g, and preterm birth was assigned if the neonate was born at < 37 completed weeks of gestation. Gestational age was determined by the date of the last normal menstrual period and by early ultrasound. Ultrasound performed in the first trimester is considered to provide a more reliable estimate, i.e., plus or minus one week, and mothers who did not know the date of their last menstrual period and had no reliable early ultrasound were excluded from the study^15^. Newborn babies were weighed on a beam balance with a 30 g accuracy within 6 h of delivery.

Maternal exposure to IPV was determined through the question: ‘‘when you were pregnant for this child did your current partner or boyfriend do any of the following things to you?” With a list of potential offences as follows:

- Physical violence: slapped, pushed or shoved, hit with fist or something else that could hurt, beaten abdomen, choked or burnt on purpose, used or threatened to use a knife, gun or another weapon.
- Emotional violence: insulted, humiliated, intimidated on purpose, threatened.
- Sexual violence: forced into sexual intercourse when you did not want it, had sexual intercourse when you did not want to because of being afraid of what the partner might do, forced to do something sexual that could be found degrading or humiliating.

If the pregnant women had experienced one or more of the above forms of violence during their current pregnancy, they were considered as having been exposed to IPV during pregnancy^15^.

### Analysis

Double data entry was conducted using EPI Info 2008. Data were exported to SPSS version 20 for analysis. A bivariate logistic regression was conducted to identify covariates with associations that were significant to a level of p ≤ 0.2. Variables meeting the criterion of p ≤ 0.2 were taken forward to the multiple logistic regression stage. Odds ratios, 95% confidence intervals and p-values were computed to identify the factors associated and to determine the strength of the association. Significance at a level of p < 0.05 was considered statistically significant for the final model.

### Ethical considerations

Ethical approval was obtained from Mekelle University; College of Health Science health research ethics review committee (ethical approval number ERC 1364/2018). Next, an official letter was submitted to the Tigray Regional Health bureau and to each healthcare facility, and permission was obtained from the regional health bureau and each participating healthcare facility. Written informed consent was obtained from each study participant prior to data collection. Each participant was informed about the objectives of the study and that the study findings would contribute necessary information for policymakers and other concerned bodies. Participants were also informed that all data obtained from them would be kept confidential and all data were collected in a private area. Study participants were also informed of their full right to refuse, or withdraw from, any part or all of the study.

### Ethics approval and consent

Ethical clearance was secured from Mekelle University Institutional Review Board (IRB). The study participants provided written informed consent to participate in the study after receiving information about the purpose of the study, risks and benefits, and their rights.

### Ethics declarations

The study was conducted in accordance to relevant guidelines and regulations.

## Results

### Socio-demographic characteristics

Six hundred and forty seven participants were enrolled with a response rate of 99.8%. Out of the total participants, 458 (70.78%) of them were urban residents. A majority of the participants 530 (81.9%), were aged between 20 and 35 years although a few were younger. Most participants (N = 610; 94.28%) were married. Nearly half the participants were housewives (N = 301; 46.5%) (Table 1).

**Table 1:** Socio-demographic characteristics of participants, Tigray, North, Ethiopia, 2018.

Variable		Frequency	Percent
Residence	Urban	458	70.78
	Rural	189	29.22
Age	≤ 19	50	7.7
	20–34	530	82
	≥ 35	67	10.3
Religion	Orthodox	581	89.8
	Muslim	66	10.2
Educational status	Unable to read and write	108	16.7
	Able to read and write	44	6.8
	Primary education	175	27
	Secondary education and college	211	32.6
	Diploma and above	109	16.8
Marital status	Married	610	94.3
	Single	37	5.7
Occupational status	Housewife	301	46.5
	Merchant	71	11
	Farmer	127	19.6
	Private employee	42	6.5
	Governmental employee	95	14.7
	Others	11	1.7

### Obstetrics characteristics of the participants

Of the 647 women, 155 (24%) were delivered via cesarean section. Around one fourth (162; 25%) of the women had premature rapture of membranes (PROM), 66 (10.2%) women had hypertension during their pregnancy, and 35 (5.4%) women had antepartum hemorrhage (APH). Of all women delivered in the study hospitals, 611 (94.4%) had ante natal care (ANC) follow-up for their last baby. Forty-two (6.5%) women reported the pregnancy as being unwanted. Seventy (10.8%) women delivered before term and 120 (18.5) babies were of low birth weight. The number of stillbirths in this study was 23 (3.6%). From the total neonates delivered, 82 (12.7%) had a low APGAR (Appearance, Pulse, Grimace response, Activity, and Respiration) score (less than 7) (Table 2).

**Table 2:** Obstetric characteristics of participants, Tigray, North, Ethiopia, 2018.

Variable		Frequency	Percentage
Mode of delivery	Vaginal	492	76
	C/S	155	24
PROM	Yes	162	25
	No	485	75
PIH	Yes	66	10.2
	No	581	89.8
APH	Yes	35	5.4
	No	612	94.6
ANC follow up	Yes	611	94.4
	No	36	5.6
Still birth	Yes	23	3.6
	No	624	96.4
APGAR score	Less than 7	82	12.7
	greater than or equal to 7	565	87.3
Pregnancy wanted	Yes	605	93.5
	No	42	6.5
Preterm birth	Yes	70	10.8
	No	577	89.2
Low birth weight	Yes	120	18.5
	No	527	81.5

### Habits of women

Of the sample, 288 (44.5%) women, had consumed alcohol sometimes during their pregnancy, 10 (1.6%) had chewed chat, and three (0.5%) women smoked.

Regarding the habits of the father of the baby, 19 (2.9%) had drunk alcohol throughout the pregnancy and 362 (56%) had drunk sometimes. 31 (4.8%) men chat chewed sometimes, 4 (0.6%) had chewed throughout. Concerning smoking, 22 (3.4%) men smoked sometimes and 3 (0.5%) men had smoked throughout.

### Types of intimate partner violence

Of the total study participants 47 (7.3%) had experienced intimate partner violence during their pregnancy. Of these forty-seven who had experienced intimated partner violence, 22 (46.8%) had experienced physical violence, 8 (17%) psychological violence, and 39 (60%) sexual violence.

### Factors associated with low birth weight and preterm birth

Factors exhibiting associations with preterm birth (p ≤ 0.2) in the bivariate regression were hypertension, area of residence, APH, PROM, and ANC follow-up. These factors were exported to the multivariate regression. In the multivariate regression, APH, PROM, and ANC follow-up had significant associations (p < 0.05).

Factors exhibiting associations (p ≤ 0.2) in the bivariate regression with low birth weight were whether the pregnancy was wanted, ANC follow-up, and IPV during pregnancy, and these factors were exported to the multivariate regression. In the multivariate regression, only IPV during pregnancy was significantly associated with low birth weight (p ≤ 0.05).

Women who had had experienced intimate partner violence during pregnancy had 2.39 (95% CI: 1.26–4.55) times the risk of delivering a low-birth-weight baby compared to women who had not experienced violence. Women who had no ANC follow-up, APH, or PROM were significantly more likely to have a preterm birth. Women who had no ANC follow up had 3.9 (95% CI: 1.8–8.6) times the risk of preterm birth compared to their counterparts. Similarly, women who had PROM had 1.86 (95% CI: 1.09–3.18) times the risk of preterm birth, and women who had APH had 2.6 (95% CI: 1.1–6.1) times the risk of preterm birth compared to women who had no APH (Table 3).

**Table 3:** Bivariate and multivariate logistic regression analyses of associations between preterm birth and low birth weight with socio demographic variables, obstetric variables, and intimate partner violence during pregnancy.

Variables		Preterm birth		COR (95% CI)	AOR (95%CI)	Low birth weight		COR (95% CI)	AOR(95%CI)
		Yes	No			Yes	No		
Marital status	Marriage	68	542	1:00		113	497	0.97 (0.41–2.2)	
	Single	2	35	0.45 (0.1–1.9)		7	30	1:00	
Residence	Urban	42	416	1:00	1:00	80	378	1:00	
	Rural	28	161	1.72 (1.03–2.8)	1.59 (0.9–2.7)	40	149	1.26 (0.83–1.93)	
Religion	Orthodox	63	518	0.97 (0.41–2.2)		111	470	1:00	
	Muslim	7	59	1:00		9	57	0.66 (0.32–1.39)	
Age	> 19	6	44	1:00		10	40	0.96 (0.38–2.4)	
	20–35	56	474	1.1 (0.52–2.5)		97	433	1.07 (0.56–2)	
	> 35	8	59	0.99 (0.32–3)		13	54	1:00	
IPV	Yes	7	40	0.67 (0.28–1.56)		16	31	2.46 (1.29–4.66)	2.39 (1.26–4.55)
	No	63	537	1:00		104	496	1:00	1:00
Hypertension	Yes	4	62	1.9 (0.7–5.6)	2.2 (0.75–6.7)	13	53	1.08 (0.57–2)	
	No	66	515	1:00	1:00	107	474	1:00	
APH	Yes	9	26	3.12 (1.4–6.9)	2.6 (1.1–6.1)	9	26	1.56 (0.7–3.4)	
	No	61	551	1:00	1:00	111	501	1:00	
PROM	Yes	26	136	1.9 (1.13–3.2)	1.86 (1.09–3.18)	32	130	1.1 (0.7–1.7)	
	No	44	441	1:00	1:00	88	397	1:00	
Habit of alcohol intake	Never	36	323	0.83 (0.5–1.36)		69	290	1:00	
	Sometimes	34	254	1:00		51	237	0.9 (0.6–1.35)	
Pregnancy wanted	Yes	65	540	0.89 (0.33–2.3)		108	497	1:00	1:00
	No	5	37	1:00		12	30	1.8 (0.9–3.7)	1.5 (0.7–3.4)
ANC follow up	Yes	59	552	1:00	1:00	110	501	1:00	1:00
	No	11	25	4.1 (1.9–8.7)	3.9 (1.8–8.6)	10	26	1.75 (0.82–3.7)	1.3 (0.6–3.2)

## Discussion

This study aimed to determine whether intimate partner violence during pregnancy was associated with preterm birth and low birth weight. Intimate partner violence during pregnancy was not found to be significantly associated with preterm birth, but there was a significant association with low birth weight.

The prevalence of intimate partner violence during pregnancy was 7.3%. The findings of this study are supported by the findings of a previous data analysis conducted across 19 countries, that reported the prevalence of intimate partner violence to range from 4 to 13.5%^16^. The present study reports a higher prevalence than that of a study (3.7%) conducted in New York City^17^. The difference may be attributable to New York having a better level of gender equality than that present in Ethiopia. It has been reported that if there is gender equality, intimate partner violence will be less. However, the prevalence of intimate partner violence reported in the present study is lower than that reported in Tanzania (30%), Vietnam (32.5%), and Ethiopia Hosanna (23%)^11–13^. This might due to recent measures taken by the Ethiopia government in their second Growth and Transformational Plan, that prioritized ending violence against women^4^. The disparities between the present study and those conducted in Vietnam and Tanzania are likely due to differences in the study methodologies used in Tanzania and Vietnam, as the present study used a cohort study design.

In this study, the prevalence of preterm birth was 10.8% and the prevalence of low birth weight was 18.5%. A study from Kenya reported a higher rate of preterm birth (18.3%) compared to this study^18^. Conversely, a study conducted in Vietnam reported a 6.2% prevalence of preterm birth and a 4.9% prevalence of low birth weight^11^. A study conducted in Baltimore, Maryland, reported a prevalence of low birth weight of 21%^19^. These variations could be due to the prevalence of preterm birth and low birth weight varying with different environments, healthcare systems, and advice given during pregnancy, all of which are likely to be associated with increased or decreased risk of preterm birth and low birth weight.

This study found that IPV during pregnancy had a significant association with low birth weight. This finding is in line with a study conducted in Vietnam, which reported that experiencing physical violence was associated with an increased the risk of low birth weight^11^. Similarly, in a study from Tanzania, women exposed to physical violence were found to be three times more likely to give birth to a baby with low birth weight^12^. A further study conducted in low income women has shown IPV during pregnancy to be associated with a four-fold increase in low birth weights^10^.

These findings also agree with a study conducted in Ethiopia, where the findings indicated that women experiencing IPV during pregnancy had an increased risk of low birth weight^13,14^.

Multiple studies have demonstrated IPV to have a significant association with low birth weight. This may be due to the effect of IPV on the woman having both physical and mental components, and as such, low birth weights can occur either by direct or indirect mechanisms.

In this study, IPV had no association with preterm birth. However, women who had no ANC follow up, PROM, or APH did have a significantly higher risk of preterm birth. Similar findings have shown that women without ANC follow up have four times the risk of preterm birth as compared to their counterparts^20^. Research conducted in Tanzania has shown that women who lacked ANC follow up had five times the risk factor for delivering prematurely^21^. This finding may be related to ANC including valuable counseling, covering health education about nutrition and disease prevention and health promotion. It is likely that receiving this counseling may be contributed to the prevention of preterm birth.

Again, women with PROM have nearly twice the risk of preterm birth. A study conducted in Iran provides further confidence in this result, whereby women who experienced PROM had three times the risk factor for preterm birth^22^. Similarly, research findings from Kenya reported that women with PROM were at a fivefold risk for preterm birth^18^. These findings may be associated with a common need for the induction of labor secondary to chorioamnionitis. Microorganisms that cause bacterial vaginitis can simply ascend and cause intrauterine infections. It is assumed that subclinical chorioamnionitis may trigger the release of inflammatory mediators which lead to release of prostaglandins from the uterine decidua that ultimately induce preterm labor.

Finally, APH increased the risk of preterm birth by a factor of nearly three. This finding is supported by research conducted in Iran, whereby women with bleeding during pregnancy had double the risk factor for preterm birth^22^, and research conducted in Kenya showed that having APH increased the risk of preterm birth four-fold^18^. The causes for vaginal bleeding during the first half of pregnancy are unknown. However, much of the time, bleeding during the second or third trimester of pregnancy is attributed to placenta previa or placenta abruption. Vaginal bleeding may lead to emergency treatment for the fetus or mother and may in turn induce preterm birth.

### Limitation of the study

This study did not assess the frequency of intimate partner violence experienced by each participant, and therefore associations with the frequency and severity of violence could not be addressed.

## Conclusion

This study demonstrated that intimate violence during pregnancy is high and that intimate partner violence during pregnancy significantly associated with an increased risk of low birth weight.

### Recommendation and policy implication

The federal minster of health should introduce measures to prevent intimate partner violence during pregnancy to reduce adverse birth outcomes.

Intimate partner violence screening should be involved at ANC.

Researchers should conduct a case–control study to see if there is a measurable cause and effect between intimate partner violence during pregnancy and low birth weight / preterm birth.

## Data Availability

The data supporting the findings of this article can be made available on request.
